# Potential Effects of Mina53 on Tumor Growth in Human Pancreatic Cancer

**DOI:** 10.1007/s12013-014-9841-7

**Published:** 2014-02-13

**Authors:** Xiao-ping Tan, Wei-guo Dong, Qing Zhang, Zi-rong Yang, Xiao-fei Lei, Ming-hua Ai

**Affiliations:** 1 Department of Gastroenterology, No. 1 Hospital, Yangtze University, Jingzhou, 434000 Hubei People’s Republic of China; 2 Department of Gastroenterology, Renmin Hospital, Wuhan University, Wuhan, 430060 Hubei People’s Republic of China

**Keywords:** Mina53, Pancreatic cancer, RNA interference, Quantum dots, Tumor growth

## Abstract

Myc-induced nuclear antigen (Mina53) is a protein with a molecular weight of 53 kDa expression of which is induced by c-Myc. Increased expression of Mina53 is documented in some human carcinomas. In this study, we found markedly increased Mina53 expression in pancreatic cancer tissue specimens. This expression did not correlate with clinicopathological characteristics, such as sex, age, and presence of distant metastasis. However, there was a statistically significant association with histological differentiation, TNM stage, and lymph node metastases. To study functional role of Mina53, we silenced its expression by siRNA in PANC-1 cells. These cells were arrested in the G2/M phase, and apoptosis rates were increased. In conclusion, increased expression of Mina53 may play an important role in the development of human pancreatic cancer. Mina53 can be used as a marker for pancreatic cancer and may potentially be exploited as a target for treatment of pancreatic cancer.

## Introduction

Pancreatic cancer is a highly aggressive and deadly disease. Worldwide, more than 250,000 people die annually of pancreatic cancer [1]. Pancreatic cancer refers to a malignant neoplasm originating from transformed cells resident to pancreatic tissues. The most common type of pancreatic cancer, which accounts for 95 % of these tumors, is adenocarcinoma, which is a tumor exhibiting glandular architecture on light microscopy. Adenocarcinomas arise from the exocrine component of the pancreas [2]. A minority of tumors arises from islet cells and is classified as neuroendocrine tumors. Most patients are diagnosed at an advanced stage of the disease, and the incidence and mortality rates for invasive pancreatic cancer are almost identical [3]. Treatment has not improved significantly during the past few decades and is still insufficiently effective in increasing survival of these patients [4–6]. Therefore, a better understanding of carcinogenesis of pancreatic cancer and its prevention are recognized as important factors in reducing mortality worldwide.

The Myc family of proto-oncogenes consists of three main genes: c-Myc, N-Myc, and L Myc [7]. Abnormal expression of Myc family genes has long been known to be associated with neoplastic diseases in a wide range of vertebrates, including humans. Besides cancerogenesis, the genes of the Myc family are also involved in many other biological processes. Specifically, in addition to cell proliferation, these genes also control apoptosis and cell differentiation. c-Myc is one of the most widely studied proto-oncogenes. In general, its increased expression is associated with cell proliferation, while the expression is down-regulated in quiescent and differentiated cells.

The proteins encoded by the Myc family genes are members of the basic helix-loop-helix leucine zipper transcription factors and appear to control expression of several other genes that mediate each of the Myc functions. However, unidentified Myc target genes may still exist, and the mechanisms by which Myc contributes to tumorigenesis are still not fully understood [8–15].

Myc-induced nuclear antigen (Mina53) is a protein with a molecular weight of 53 kDa expression of which is directly induced by c-Myc [16, 17]. The protein is predominantly localized in the nucleus, although some of the protein is also expressed in the nucleolus [16–19]. Expression of Mina53 is increased during cell proliferation, and specific inhibition of Mina53 expression by RNA interference (RNAi) suppresses cell proliferation [18]. Recently, a monoclonal antibody against human Mina53 protein was generated. Using this antibody, increased expression of Mina53 was documented in some human carcinomas [18–27], such as colon carcinoma and esophageal squamous cell carcinoma. These data suggest that Mina53 may be associated with carcinogenesis.

In the present study, we evaluated expression of Mina53 in pancreatic ductal adenocarcinomas and further studied the relationship between its expression and clinicopathological characteristics of human pancreatic cancer. Further, using RNAi, we evaluated the role of Mina53 in the carcinogenesis and progression of tumor to test the potential clinical usefulness of Mina53 as target for anti-cancer therapies.

## Materials and Methods

### Tissue Microarray (TMA)

TMA was constructed from a series of paraffin-embedded specimens including 96 cases of pancreatic ductal adenocarcinomas and 34 specimens of normal pancreatic tissue that were collected from 2006 to 2011 at the Pathology Departments of Renming Hospital of Wuhan University and Jingzhou First People's Hospital in China. Tissue specimens were arranged in 20 columns and 12 rows for a total of 200 individual cores (1.1 mm and 4 μm). Each sample had two replicates on the TMA. Each slide had more than 95 % tissue core retention. Clinicopathological characteristics of 96 pancreatic cancer cases were as follows: mean age 49.2 years (range 32–80), 61 male and 35 female patients, 28 cases of stage I, 43 cases of stage II, 15 cases of stage III, and 10 cases of stage IV according to pathological TNM staging.

### Quantum Dot-Based Immunofluorescence Histochemistry (QDs-IHC)

The 4-μm thick TMAs were deparaffinized in xylene and rehydrated in graded ethanol. QDs-IHC was performed according to the manufacturer’s instructions (Wuhan Jiayuan Quantum Dot Co LTD, Wuhan, China). Antigen retrieval was performed in citric acid (10 mM and pH 6.0) at 95 °C for 10 min, followed by cooling for 30 min. For antibody binding, TMAs were first incubated with 2 % bovine serum albumin (BSA) buffer (Sigma, St. Louis, USA) for 30 min at 37 °C followed by overnight incubation at 4 °C with primary mouse anti-human Mina53 monoclonal antibody (dilution 1:100, Invitrogen, San Francisco, USA). TMAs were then washed 3 × 5 min with TBS-T (0.5 % Tween, 0.1 M Tris-base, and 0.9 % NaCl; pH 7.6) and incubated with biotinylated goat anti-mouse IgG (dilution 1:100, Jackson ImmunoResearch, West Grove, USA) for 30 min at 37 °C. Negative control samples were prepared in parallel by replacing primary antibody with TBS. For QDs conjugation, antibody-binding TMAs were again incubated in 2 % BSA buffer for 10 min at 37 °C, incubated with QDs (605 nm), and conjugated to streptavidin (QDs-SA) (dilution 1:200 in 2 % BSA, Wuhan Jiayuan Quantum Dot Co LTD, Wuhan, China) for 30 min at 37 °C. The specimens were rinsed 3 × 5 min with TBS-T and sealed with 90 % glycerine (Sigma-Aldrich, St. Louis, USA).

QD signals were detected using Olympus BX51 fluorescence microscope equipped with Olympus Micro DP 72 camera (Olympus Corporation, Tokyo, Japan). Signals were red, bright, target specific, and photostable. At least one hundred cells from five representative fields of each core were randomly selected and counted blindly by two independent observers using a 40× objective. Mina53 expression was assessed semi-quantitatively and classified into three groups on an arbitrary scale of 0, 1, and 2 [28]. Specifically, percentage of positively stained tumor cells and staining intensity were recorded for each sample. A value of zero was assigned to specimens with fewer than 10 % positive cells, 1 represented weak homogeneous staining, and 2 was given to specimens with intense staining.

### Cell Culture

Human pancreatic cancer cell line PANC-1 was maintained in RPMI 1640 (Invitrogen) supplemented with 10 % fetal bovine serum, 100 units/ml penicillin and 100 μg/ml streptomycin in a humidified incubator at 5 % CO_2_ and 37 °C.

### Cell Transfection

Mina53 siRNAs were designed using BLOCK-iT™ RNAi Designer (Invitrogen) using accession number from Gene bank. siRNA were synthesized by Jima Bio. Corp. (Shanghai, China). The target sequences were as follows: sense 5′-GCCGGAUCAAGAUCAAUCUTT-3′ and antisense 5′-AGAUUGAUCUUGAUCCGGCTT-3′. Negative control siRNA (i.e., nontargeting siRNA) was purchased from Jima Bio. Corp.

PANC-1 cells were seeded onto collagen I-coated six-well plates at cell density of 5 × 10^4^ cells/well and cultured overnight. The following day, cells were transferred in serum-free medium and transfected with siRNA using Lipofectamine 2000 (Invitrogen) for 4 h at 5 % CO_2_/37 °C. After 4-hour incubation, an equal volume of medium supplemented with 20 % FBS was added to wells, and transfected cells were cultured until further analyses.

### Proliferation Assays

Cells were transiently transfected with Mina53 siRNA and counted using trypan blue staining. After about 5 × 10^3^ cells were cultured, the number of cells was counted at indicated time points. The experiments were repeated three times.

### Real-Time RT-PCR

The levels of Mina53 mRNA after transfection were quantified by real-time RT-PCR. Total RNA was isolated from PANC-1 cells using TRIzol (Invitrogen). RNA concentration was determined using NanoDrop 2000 Spectrophotometer. 1 μg total RNA was reverse transcribed with MuLV reverse transcriptase (Roche Molecular Systems Inc., Branchburg, USA) at 37 °C for 60 min. The cDNAs were utilized in SYBR Green real-time PCR. Each 20 μl of PCR reaction contained 2 μl cDNA, 2 μl 10 × LightCycler-DNA Master Mix SYBR Green I (Roche Diagnostics Corp., Indianapolis, USA), and 1 μM forward and reverse primers. The primer sets were as follows: 5′-CCCATTATGATGATGTCGA-3′ (sense) and 5′-TGTTCTGGTAGGTGCTGAT-3′ (antisense). Real-time PCR was performed using the LightCycler™ V3 System (Roche Diagnostics Corp). The PCR conditions were as follows: 35 cycles of 2 s each at 95 °C, 10 s at 55 °C, and 15 s at 72 °C. Expression of β-actin mRNA was used as control.

### Western Blot

To detect protein level of Mina53 after transfection, PANC-1 cells were treated with siRNA as above, washed with PBS, and lysed in RIPA buffer containing protease inhibitor cocktail. The lysates were centrifuged and supernatants collected. Protein concentrations were determined using Bio-Rad Protein Assay (Bio-Rad, Hercules, USA). Protein extracts (40 μg) were separated on 12.5 % SDS-PAGE. The proteins were transferred onto nitrocellulose membranes. The membranes were blocked using 5 % nonfat dry milk and incubated overnight at 4 °C with primary antibodies (anti-Mina53 or anti-actin; both from Santa Cruz Biotechnology, Santa Cruz, CA, USA), followed by incubation with HRP-conjugated secondary antibodies for 1 h at room temperature. Membranes were developed using ECL Substrate (Amersham Corp., Arlington Heights, USA). Protein bands were visualized on X-ray film.

### Cell Cycle and Apoptosis Analysis

The analysis of cell cycle was performed as follows. At indicated times after transient transfection of PANC-1 cells with Mina53 siRNA, floating cells were collected by pipetting and adherent cells by trypsinization. Both cells were combined and fixed with 2 % paraformaldehyde followed by permeabilization with 70 % ethanol. Cells were treated with RNase A (0.25 mg/ml) at 37 °C for 30 min and stained with propidium iodine (50 μg/ml). Cellular DNA content was analyzed using flow cytometer (BD Biosciences, Franklin Lakes, USA). Cell cycle profiles were determined with CELLQuest™ software (BD Biosciences). For apoptosis analysis, cells were simultaneously stained with Annexin V and propidium iodide kit (BD Biosciences) according to the manufacturer’s instructions. Stained cells were analyzed by flow cytometry to quantify the number of apoptotic cells.

### Statistical Analyses

For data compilation and statistical analysis, the software package SPSS version 13.0 was used (SPSS Inc, Chicago, USA). The Chi-square and Fisher’s exact probability tests were used to examine associations between Mina53 expression and various other parameters including clinicopathological characteristics. Number of colonies was compared between PANC-1 cells transiently transfected by Mina53 siRNA using the Student’s *t* test. Statistical significance was assumed at a *P* value < 0.05.

## Results

### Mina53 Expression in Normal Pancreatic Tissue and Pancreatic Ductal Adenocarcinoma

**Fig. 1:**
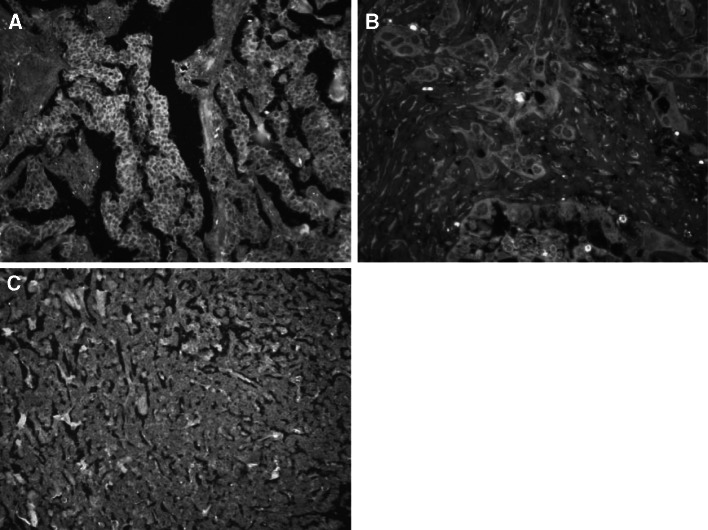
Expression of Mina53 in TMA of pancreatic cancer and normal pancreatic tissue specimens. **a** Positive expression in pancreatic cancer tissue (×200). **b** Positive expression in pancreatic cancer tissue (×400) **c** Negative expression in normal pancreatic tissue (×200)

Positive Mina53 staining was mainly localized in nuclei and, to a certain degree, in cytoplasm (Fig. 1). Normal pancreatic specimens showed positive staining for Mina53 in only two out of 34 cases (5.9 %). By contrast, overexpressed Mina53 was found in 81 of the 96 adenocarcinoma specimens (84.4 %; Table 1).

**Table 1:** Mina53 expression in human normal pancreatic tissue and pancreatic ductal adenocarcinoma

Groups	Total	Negative	Positive	Cases with positive staining
Normal pancreatic tissue	34	32	2	3 (5.9 %)
Pancreatic ductal adenocarcinoma	96	15	81	83 (84.4 %)

Expression of Mina53 in tumor specimens was compared to expression in normal pancreatic tissue. Mina53 expression was significantly higher in adenocarcinomas compared with normal specimens (*P* < 0.01, Table 1).

### Relationship Between Mina53 Expression and Clinicopathological Characteristics of Pancreatic Cancer

There were no significant associations between Mina53 overexpression and clinicopathological characteristics, such as sex and age (Table 2). By contrast, there was a statistically significant association with histological differentiation (*P* < 0.05), TNM stage (*P* < 0.01), and lymph node metastasis (*P* < 0.05). Significant associations between Mina53 and distant metastasis were not found (Table 2).

**Table 2:** Relationship between expression of Mina53 and clinicopathological characteristics of pancreatic cancer

Clinical characteristics	Total	Mina53		*P*
		Negative	Positive	
All cases	96	15	81	
Sex				
Male	61	8	53	0.371
Female	35	7	28	
Age				
≤60 years	49	9	40	0.450
>60 years	47	6	41	
Histological differentiation				
Well	28	8	20	0.025
Moderate	39	5	34	
Poor	29	2	27	
TNM stage				
I	11	6	5	0.004
II	43	6	33	
III	32	2	30	
IV	10	1	9	
pN (lymph node metastasis)				
pN0	22	7	15	0.017
pN1	74	8	66	
Distant metastasis				
M0	86	15	71	0.354
M1	10	0	10	

### Effect of Mina53 siRNA Silencing on Expression of Mina53

**Fig. 2:**
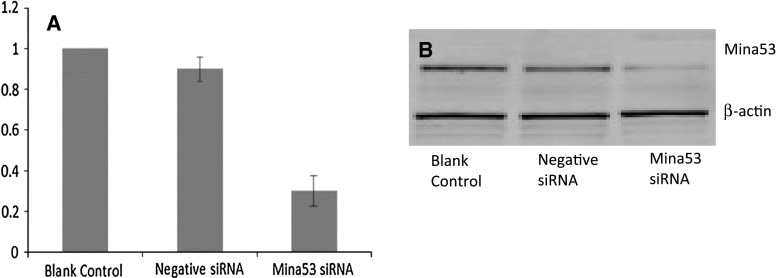
Expression of Mina53 after siRNA silencing. **a** Expression of Mina53 was down-regulated by siRNA. Relative expression was calculated as equivalent ratio (mRNA levels of the gene of interest corrected for β-actin mRNA) using the expression in blank control group as control. The *dotted line* (relative expression = 1) indicates expression level in blank control group. Data are presented as mean ± SD of three experiments. **b** The Western blot analysis confirmed diminished expression of Mina53 protein

Human Mina53 siRNA and negative control siRNA were successfully constructed as confirmed by sequencing. Pancreatic cancer cells (PANC-1) were transfected with siRNA. The siRNA silencing led to a decrease of Mina53 mRNA levels of 35.1 ± 2.7 % (*P* < 0.01 vs. blank control group; Fig. 2a). By contrast, no significant difference was found in Mina53 mRNA levels between negative siRNA and blank control groups (Fig. 2a). Further, diminished expression of Mina53 in Mina53 siRNA group was confirmed by Western blotting (Fig. 2b).

### Mina53 siRNA Silencing Diminishes Cell Proliferation

**Fig. 3:**
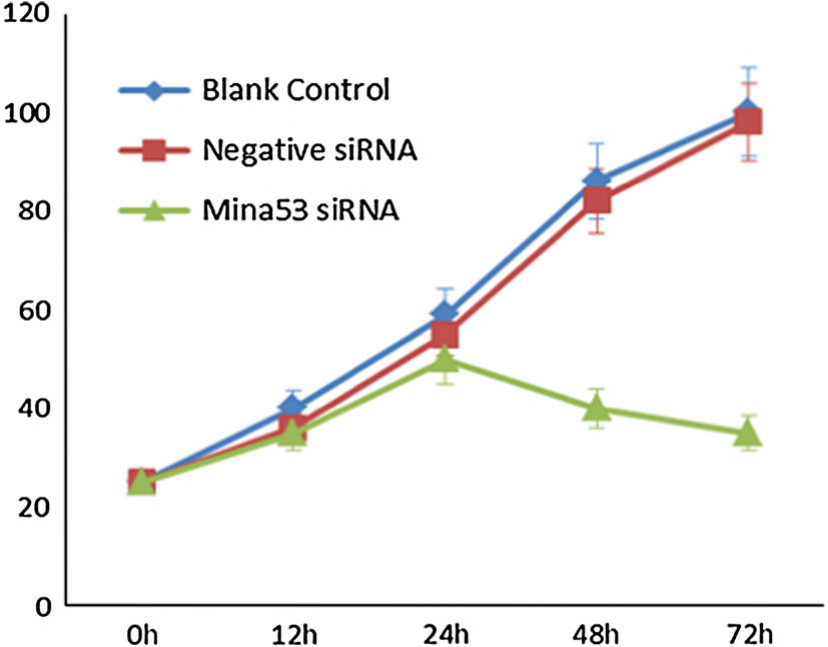
Proliferation of pancreatic cancer cells after Mina53 siRNA silencing. After 48 and 72 h post-transfection, cells treated with Mina53 siRNA exhibited significantly diminished growth rates compared with blank control cells (no siRNA) or cells transfected with negative siRNA

To further analyze the role of Mina53 in carcinogenesis, we examined the effect of Mina53 siRNA silencing on pancreatic cancer cell proliferation. After 72 h post-transfection, cells treated with Mina53 siRNA exhibited significantly diminished growth rates compared with blank control cells (no siRNA) or cells transfected with negative siRNA. As shown in Fig. 3, negative siRNA had a minimal effect on proliferation of PANC-1. By contrast, silencing of Mina53 significantly suppressed proliferation in PANC-1 cells (Fig. 3). After 48 and 72 h post-siRNA, there was an inhibition of the growth of PANC-1 cells (respectively, 40 and 35 %, *P* < 0.01; Fig. 3).

### Mina53 siRNA Induces Apoptosis

Cell cycle distribution and apoptosis after transfection were detected by flow cytometry. There was no significant difference in cell cycle distribution between blank control and negative siRNA groups (Table 3). Apoptosis rates in both groups were low.

**Table 3:** Cell cycle distribution and apoptosis after 48 and 72 h of transfection (%)

Groups	48 h				72 h			
	Apoptosis rate	G_0_/G_1_	S	G_2_/M	Apoptosis rate	G_0_/G_1_	S	G_2_/M
Blank control	0.7 ± 0.4	56.9 ± 3.5	32.2 ± 2.9	15.2 ± 1.9	0.8 ± 0.6	51.5 ± 2.4	30.8 ± 2.3	14.7 ± 2.1
Negative siRNA	0.8 ± 0.5	55.2 ± 2.8	33.7 ± 3.5	16.4 ± 1.8	0.9 ± 0.8	50.7 ± 2.7	31.7 ± 2.6	15.6 ± 1.9
Mina53 siRNA	19.3 ± 2.8	40.9 ± 3.2	19.9 ± 2.9	25.1 ± 2.8	38.9 ± 2.9	26.3 ± 2.6	9.2 ± 2.9	30.1 ± 2.5

Twelve hours post-transfection, cell cycle distribution, and apoptosis in Mina53 siRNA group were similar to blank control group (Table 3). Although apoptosis was not apparent, the distribution of cell cycle began to change, such that the proportion of cells arrested in G2 phase declined while that of cells in M phase increased (Table 3). Cell cycle distribution and apoptosis of Mina53 siRNA group at 48 and 72 h post-transfection demonstrated that the proportion of cells arrested in G2/M phase was about 1.5 times higher than in blank control group, while apoptosis rates were, respectively, 19.3 ± 2.8 and 38.9 ± 2.9 % (*P* < 0.05 Mina53 siRNA vs. blank control or negative siRNA groups; Table 3).

## Discussion

Tsuneoka et al. [16, 17] previously isolated Mina53 as a Myc target gene and showed a clear relationship between Mina53 expression and cell proliferation. Immunohistochemistry also revealed overexpression of Mina53 in gastric cancer, colon cancer, esophageal cancer, lymphoma, renal cell carcinoma, and neuroblastoma [18–27]. Mina53 expression is inversely correlated with patient survival in esophageal cancer, renal cell carcinoma, and neuroblastoma. These results suggested that Mina53 may be involved in carcinogenesis and tumor progression which led us to hypothesize that Mina53 may be involved in abnormal cellular growth in pancreatic cancer.

Pancreatic cancer is one of the most aggressive malignancies with a very poor prognosis, partially due to a very difficult accessibility to resection and resistance to chemoradiotherapy [29]. As such, it is imperative to find more effective and specific therapies [30, 31]. Identification of specific targets can be done by RNA interference (RNAi) [32–34]. As a powerful tool to suppress gene expression in mammalian cells, the RNAi can be directed against pancreatic cancer through various pathways, including the inhibition of overexpressed oncogenes, suppression of tumor growth, metastasis, and enhancement of apoptosis [35, 36].

TMA allows researchers to investigate multiple specimens simultaneously using immunohistochemistry (IHC). Quantum dots (QDs) are semiconductor nanocrystals with a core/shell structure and a large spectral band gap that possess unique photodynamic properties such as size-tunable symmetric emission bands, strong light absorbance, high fluorescent intensity, and high photostability. The QD fluorescence can be separated from background autofluorescence in biological specimens, such as cells and tissues [37–44]. Many studies of molecular targeted imaging of cancer cells and molecules demonstrated the advantages of QD-IHC, such as supreme fluorescent efficiency, better signal clarity, and a higher sensitivity and accuracy compared with conventional immunohistochemistry techniques. Therefore, QD-IHC allows to gain better insights into tumor biology [45]. Based on these considerations, we used mouse anti-human Mina53 monoclonal antibody to study the expression of Mina53 in TMA of pancreatic cancer by QD-IHC. In this study, staining quality of images by QDs-IHC was significantly higher than conventional IHC. QD-IHC in our study showed that most of pancreatic cancer specimens exhibited elevated expression of Mina53. By contrast, only two normal pancreatic specimens showed weakly positive staining.

We observed that Mina53 expression is associated with lymph node metastasis, histological differentiation, and TNM stage. Therefore, Mina53 may play some role in pancreatic carcinogenesis and can thus be used as a marker for pancreatic cancer.

The siRNA silencing of Mina53 inhibited cell growth in a human colon cancer cell line SW620, and two human esophageal squamous cell carcinoma cell lines TE-9 and TE-11, demonstrating that Mina53 plays an important role in cell growth [18, 19]. Increased expression of Mina53 is a characteristic feature in some cancer cells, therefore, targeting Mina53 may have therapeutic applications. In our study, Mina53 siRNA silencing successfully diminished pancreatic cancer cell growth. Thus, after 48 and 72 h of siRNA treatment, Mina53 siRNA inhibited the growth of PANC-1 cells by, respectively, 40 and 35 %. In addition, the silencing caused cell arrest in G2/M phase and induced apoptosis. This indicates that Mina53 gene may be one of the key factors involved in formation and development of pancreatic cancer. Therefore, Mina53 holds a promising therapeutic potential as a future treatment for pancreatic cancer and provides a theoretical basis for gene therapy of pancreatic cancer.

In conclusion, Mina53 is overexpressed in pancreatic cancer and is associated with cancer proliferation. Mina53 plays an important role in the carcinogenesis and development of pancreatic cancer. This protein can be used as a marker for pancreatic cancer and a target for treatment.
